# A commentary on ‘Approved and investigational fluorescent optical imaging agents for disease detection in surgery’

**DOI:** 10.1097/JS9.0000000000000799

**Published:** 2023-10-04

**Authors:** Si-Un Frank Chiu, Chao-Ming Hung, Chong-Chi Chiu

**Affiliations:** a Department of Computer Science; b Department of Economics, Brown University, Providence, Rhode Island, USA; c Department of General Surgery; d Department of Medical Education and Research, E-Da Cancer Hospital; e School of Medicine, College of Medicine, I-Shou University, Kaohsiung, Taiwan

*Dear Editor,*

Rehman *et al*.^1^ illuminate the present landscape of molecular imaging and sensing methods available for surgical guidance. It delves into the critical attributes of nuclear, optical, and multimodal approaches, offering insights into their strengths, limitations, and clinical applications, which should raise the awareness of related clinical professionals.

A complete excision of cancerous tissue with tumor-free specimen margins needs to consider preserving neighboring structures to minimize functional disability. The histopathological assessment of eradicated specimens is the gold standard for surgical margin examination. Still, assessing the entire surgical margin intraoperatively through histopathology in many oncological surgeries is impractical. Particular frozen section assessment of suspicious regions is achievable but laborious and subject to sampling errors, leading to a high false-negative rate. Therefore, pursuing an extraordinarily exact, immediate intraoperative way for tumor margin identification is necessary.

Fluorescence-guided surgery (FGS) is a susceptible and immediate intraoperative imaging method that assists surgeons in locating and removing malignant lesions across various clinical scenarios. Although phase 1 and 2 clinical trials of FGS currently assess its diagnostic performance, the need for objective criteria makes evaluating the clinical benefit of FGS challenging^2^. One key measure of this imaging method’s clinical value is its impact on intraoperative decision-making. During the operation, surgeons often face the difficult choice of additional or more conservative resections. Real-time fluorescence imaging relies on selective marker perfusion and retention within the tumor area. However, tumor-induced stromal desmoplasia can interfere with marker perfusion and retention. For instance, malignancies such as oral squamous cell carcinoma within oral submucous fibrosis invariably exhibit stromal desmoplasia. Moreover, poorly differentiated cancers tend to spread as individual cells due to loss of cell cohesion – conventional imaging modalities like computed tomography and magnetic resonance imaging struggle to detect such intricate areas^3^. However, clinicians have to take the risk of underestimating tumor extensions. Investigating how this underestimation might impact pre-resection immediate fluorescence imaging explication represents a significant challenge. Consequently, the proposed recommendations should include a component related to the correlation between the findings of immediate fluorescence imaging and the pattern of tumor invasion.

Another interfering factor is that the resected specimens would experience significant shrinkage upon excision (~24.8–38.3%) and during tissue processing (around 32.4–48.8%)^3^. This inherent variability in tissue shrinkage can significantly impact the histopathological evaluation of the resected specimen margins and, consequently, the correlation investigation with pre-resection immediate fluorescence imaging consequences.

Researchers have tried to tackle these challenges through both hardware and software innovations. Many imaging systems have been invented tailored for open and endoscopic surgeries and procedures harnessing NIR-II signals (optical spectrum: 1000–1700 nm). Moreover, software enhancements, particularly those based on machine learning (ML) strategies, have further alleviated the inherent limitations of hardware systems. The role of ML is primarily employed for image enhancement and image registration^4^. For instance, ML-based image post-processing methods have operated generative adversarial networks to enhance image quality, effectively mitigating fake textures and improving resolution. Synthetic data generation techniques have been used to train deep neural networks for super-resolution imaging. Furthermore, ML has been instrumental in intraoperative lesion analysis, offering automated, objective, and swift analyses of surgical specimens and in-vivo tissue during surgery^4^.

FGS is a sophisticated procedure influenced by numerous factors, some of which are challenging to quantify, including autofluorescence, optical tissue characteristics, patient and tumor distinctiveness, pharmacokinetics, targeting specificity, and imaging systems. However, Dr Ishizawa stated that the convergence of artificial intelligence (AI) technologies with intraoperative fluorescence imaging promises to enhance the precision and dependability of FGS^5^. AI allows for developing algorithms considering relevant histopathological features alongside real-time fluorescence imaging data^3^. These imaging techniques are also expected to contribute to developing AI-driven intraoperative navigation systems. For instance, deep learning algorithms that utilize preoperative and intraoperative images can facilitate the creation of a ‘virtual’ surgical anatomy, allowing real-time assessment of the risk of organ injury during operation^5^.

The synergy between AI and intraoperative fluorescence imaging represents a remarkable advancement in surgery, offering new dimensions of accuracy and safety. It equips surgical oncologists and interventional radiologists with macroscopic and microscopic perspectives on cancer within the operating room. As optical imaging continues to evolve, clinicians will have access to an expanding array of optical modalities for clinical practice and research, propelling the next wave of surgical innovation.

## Ethical approval

This is only a commentary, not research involving patients. No ethical approval is required.

## Consent

This is only a commentary, not research involving patients. No patient consent is required.

## Sources of funding

This is a commentary. We have no funding for our commentary.

## Author contribution

S.-U.F.C.: conceptualization and writing the original draft; C.-M.H.: validation; C.-C.C.: conceptualization, supervision, submission, and correspondence.

## Conflicts of interest disclosure

There are no conflicts of interest in our commentary.

## Research registration unique identifying number (UIN)

This is a commentary on a study published in ‘International Journal of Surgery’. No UIN is required.

## Guarantor

Professor Chong-Chi Chiu.

## Data availability statement

This is only a commentary on a study published in ‘International Journal of Surgery’. There is no research data in our commentary.

## Provenance and peer review

This paper was not invited.
